# Humidity governs the wall-inhabiting fungal community composition in a 1600-year tomb of Emperor Yang

**DOI:** 10.1038/s41598-020-65478-z

**Published:** 2020-05-21

**Authors:** Yonghui Li, Zhi Huang, Evangelos Petropoulos, Yan Ma, Yang Shen

**Affiliations:** 1 School of Architecture, Southeast University, 210096 Nanjing, P.R. China; 2 Key Laboratory of Urban and Architectural Heritage Conservation (Southeast University), Ministry of Education, 210096 Nanjing, P.R. China; 3 Key Laboratory of Agricultural Environmental Microbiology, Ministry of Agriculture, College of Life Sciences, Nanjing Agricultural University, 210095 Nanjing, P.R. China; 4 School of Engineering, Newcastle University, Newcastle upon Tyne, NE1 7RU UK

**Keywords:** Fungal biology, Microbial ecology

## Abstract

Biodeterioration caused by filamentous fungi is often a threat to the architectural heritage (i.e. tombs and historic sites). To specifically understand the deterioration phenomena caused by microorganisms in tombs and how these are shaped due to various environmental factors, the fungal communities in the coffin chamber of the Chinese emperor Yang (BC 569–618) were investigated at different heights using denaturant gradient gel electrophoresis (DGGE) fingerprinting. The associated environmental conditions, such as humidity, temperature, height and illumination, were also assessed. The results showed that a great diversity of fungal species (*Cordyceps*, *Fusarium*, *Harpochytrium*, *Emericellopsis*, *Volutella*, *Cladosporium*, *Stachybotrys*, *Trichoderma*, *Cochlonema* and two unknown fungal species) was present in emperor Yang’s coffin chamber. The predominant species were *Stachybotrys*, *Fusarium*, *Trichoderma* and *Cochlonema*. Redundancy analysis (RDA) indicated that humidity, temperature, height and illumination were the most significantly related factors shaping the fungal communities. Humidity showed the highest degree of variance description (19.2%) than all other environmental factors, followed by illumination (18.3%) and height (12.8%). Furthermore, fungal richness and diversity indices showed a positive correlation with humidity (*p* < 0.05). These results help in understanding the fungal community in tombs, promoting the mitigation of deterioration phenomena of such building heritage for the present and future.

## Introduction

Brick masonry structure is the most widespread building material that accounts for a major fraction of the ancient architectural history worldwide. In China, most of historical artifacts are constructed of black brick masonry. However, the main drawback of such masonry is the fact that is prone to deterioration due to its porous and rough surface^1^. Environmental factors such as sunlight, temperature, water content, rain, wind and relative humidity also contribute to that. Physical and biophysical damages in such structures are often related to the loosening of the intergranular bonds caused by porous volume fluctuations (expansion and shrinkage). this weakens the strength of the building materials^2^ and subsequently its structure. Microbial populations, and their corresponding volume, often develop into the brick or plaster of these structures. This is also a key factor that contributes to deterioration of these structures. Fluctuations in the volume of the microbes at the developed voids in the masonry structure is also a factor that compromises the structural capacity of these buildings. A very common ‘suspect’ of such microbial/fungal fluctuations are fungi or lichen hyphae^3,4^.

A wide range of studies demonstrated that microbes could promote the biodeterioration of historical artifacts that were constructed by black bricks or stones^5,6^. As a result, deterioration usually causes an aesthetically detrimental effect^3,7^, rendering the preservation and maintenance of these valuable cultural heritage extremely challenging and financially unattainable. The porous structure of blue bricks makes such material easily adhered by various microorganism^8^; then these microorganism utilize the water and nutrition in pores to reproduce to a larger scale. Wang *et al*.^5^ isolated the bacterial strains from the surfaces of weathered bricks, and considered that *Bacillus*, *Massillia* and *Brevibacillus* could contribute to brick weathering. Vasanthakumar *et al*.^9^ assumed that the brown spots on the walls of King Tutankhamun tomb was related to microbiological activity. Their research showed that *Penicillium chrysogenum*, which could produce malic acid in vitro, was very likely responsible for the formation of these spots. However, previous research/studies only provide with a rough outline of the relationship between microorganism and building heritage deterioration, while the mechanism is not yet very clear. Therefore, we shout that there is a need to extend the knowledge on the microbial communities or the species developed on the surface of these valuable cultural heritage, so we can further understand the nature of the problem and subsequently protect our heritage for the future.

Generally, the degree of biodeterioration is dependent on the environment factors and the characteristics of the substrate (masonry)^10,11^. Porosity, surface roughness, moisture content, pH value of substrate, the mineral and nutrient concentrations all influence both microbial colonization and reproduction^12,13^, and subseqenty deterioration. Liu *et al*.^14^ reported that a biological community grows easier under conditions of water seepage and/or of high humidity. Moreover, several environment-related means are developed and/or improved to limit the biological colonization, i.e. reduction and maintenance of humidity and condensation levels^15^, effective drainage of rainfall precipitation^16^, and minimization of the moisture levels on the substrate^14^. Nevertheless, the control and the mitigation of biodeterioration processes are still far from being implemented, since the impact of the environmental factors on such sites is ignored or not thoroughly assessed. Further understanding will certainly tackle bio-deterioration of our historical heritage.

**Figure 1:**
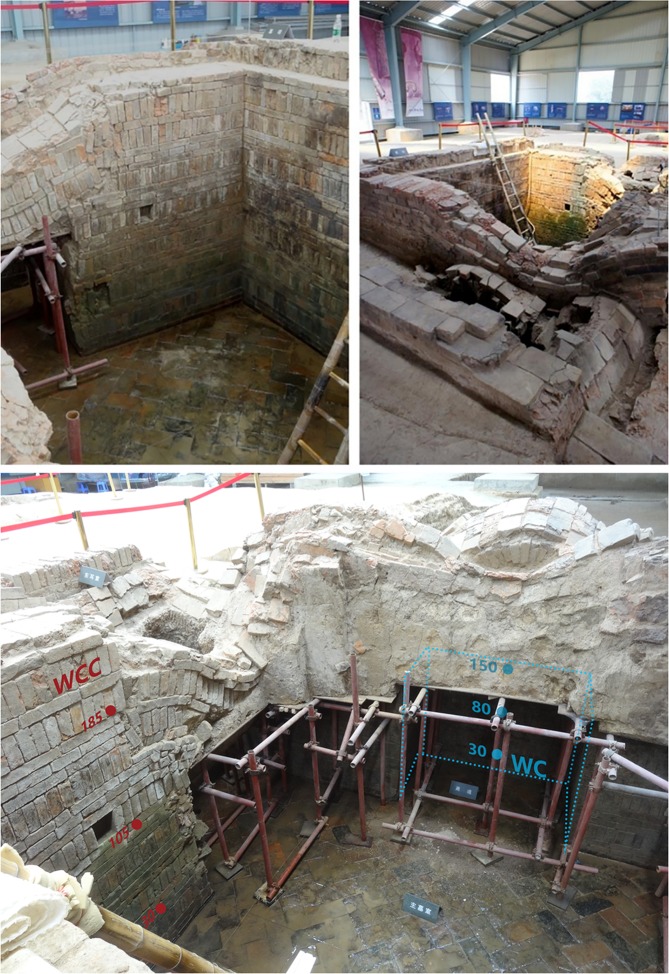
Emperor Yang’s coffin chamber and sampling sites. WC means the points on the walls of passage in the coffin chamber, 30, 80 and 150 means the three sampling points are 30, 80 and 150 centimeters above the chamber floor; WCC refers to points on the walls of main room, and the three points are 30, 105 and 185 centimeters above the floor.

**Figure 2:**
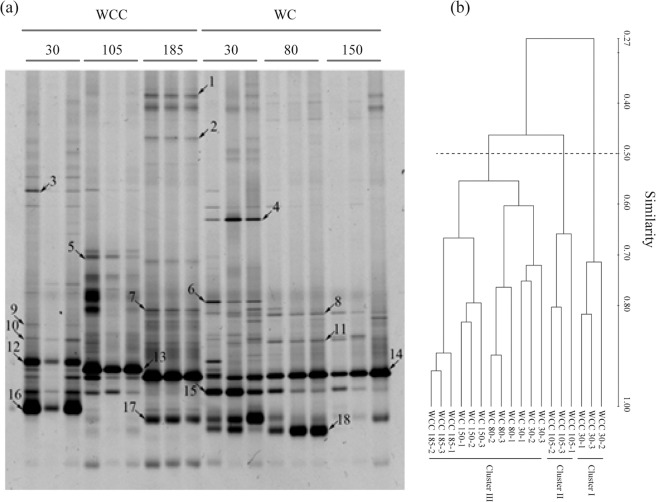
Analysis of fungal community on the walls of emperor Yang’s coffin chamber: (**a**) DGGE band patterns based on fungal partial 18S rRNA gene sequences; (**b**) Similarity of samples based on UPGMA clustering method. WC means the points on the walls of passage in the coffin chamber, and WCC means on the walls of main room. The numbers refers to the height of the sampling points are 30, 80, 150, 105 and 185 centimeters above the floor of coffin chamber.

The tomb of Emperor Yang of the Sui Dynasty, an ancient tomb with special historical and cultural value, was accidentally discovered in March 2013 at Yangzhou, China. This ancient tomb is constructed by black brick at a size of 3.45 m in width, 4.08 m in length, and 2.95 m in height (Fig. 1). As shown in Fig. 2, the tomb is currently semi-buried, and partly exposed to the natural environment. The underground part of brick masonry is exposed to high water content due to the high underground water table (two to three meters underground) present at the region of Yangzhou. Biodeterioration of this tomb became the largest worry after the initial understanding of its impact as well as the challenging need for preservation. As previously mentioned, the porosity and roughness of bricks promote microbial colonization and biofilms’ formation, account for plausibly the most complex and serious problem in conservation of architectural heritage. We hypothesized that the walls of Emperor Yang’s coffin chamber have been colonized by diverse fungal communities, and the environmental factors affected the distribution of them. In this study, the fungal community composition developed on the walls of different sections of the tomb is investigated via denaturant gradient gel electrophoresis (DGGE) fingerprinting technique. The microbial shifts along gradients of humidity, height and illumination on the walls of this tomb are also analyzed. Although microorganism colonization is hard to be controlled^10,17^, this study investigates the possibility for mitigation of such deterioration phenomena of brick masonry structure heritage, from the perspective of controlling the microorganisms that could be developed or not by manipulating environmental factors.

## Results

### Environmental characteristics in the tomb

The results of the environmental characteristics of the tomb are shown in Table 1. In this below-ground level space, the temperature and humidity of the air had an evident gradient along the vertical walls. On the passage wall, air temperature and humidity were significantly different (*p* < 0.05) along vertical direction. The temperature of WC30, WC80 and WC150 (Fig. 1), increase the higher the location with values of 24.1 °C, 25.0 °C and 25.4 °C respectively. The relative humidity were respectively 97.1%, 88.1% and 75.3%. On the wall of the main room, a similar pattern was observed. This further supports that the height increase is negatively related to the level of humidity (*p* < 0.05) and positively related to air temperature (*p* < 0.05). The illumination in all sampling sites on the passage wall were “very low”, while the assessed points on the top sites of the main wall (height 185 cm and 105 cm) were high; illumination at the underlayer (height 30 cm) was found low.

**Table 1:** Sampling sites and physical properties in the emperor Yang’s coffin chamber.

Treatments	Height (cm)	Humidity (%)	Temperature (°C)	Illumination
WCC30	30	97.03 ± 0.06a	24.07 ± 0.06c	Low
WCC105	105	88.33 ± 0.58b	24.97 ± 0.06b	High
WCC185	185	77.00 ± 0.05c	25.47 ± 0.06a	High
WC30	30	97.13 ± 0.15a	24.10 ± 0.10c	Very low
WC80	80	88.10 ± 0.36b	25.00 ± 0.00b	Very low
WC150	150	75.33 ± 0.58c	25.37 ± 0.05a	Very low

The values are the means of the three replicates (mean ± SD). Values followed by different letters differ significantly (*p* < 0.05).

### DGGE pattern and diversity in Emperor Yang’s coffin chamber

PCR-DGGE fingerprinting was conducted to reveal the shifts in fungal community composition in different positions in WCC and WC (Fig. 2a). In general, WCC30 was found the richest with regards diversity with 9 dominant DGGE bands, only 4 dominant bands were observed for WC150. The distributions of the DGGE-bands across different samples were distinct. For example, DGGE bands 14 and 15 commonly appeared in all samples; DGGE bands 1 and 2 were only detected in WCC185; DGGE band 5 was only present in WCC105; DGGE bands 4 and 6 were only found WC30. Comparing the variations of DGGE-band intensity, it is found that the highest intensities of DGGE-bands 12, 13, 14, 15, 16, 17 and 18 were respectively observed in WCC30, WCC105, WCC185, WC30, WCC30 and WC80. Besides, we also noticed that bands 1, 2, 5, 7 and 13 only appear in the positions with relatively high illumination, while DGGE-bands 3, 4, 6, 8, 11 and 18 appeared in the positions in darkness.

Cluster analysis, based on the band pattern on the DGGE gel, revealed the differences among the samples at different sites. The profiles were mainly divided into three clusters (Fig. 2b) at a similarity value of 50%. One cluster included WCC30-1, WCC30-2 and WCC30-3; the second cluster contained WCC105-1, WCC105-2 and WCC105-3; and the remaining samples formed the third cluster. According to the sampling sites, higher similarity values of samples were observed between WC30, WC80 and WC150. Comparably, samples from WCC30, WCC 105 and WCC185 showed lower similarity values. Interestingly, samples takenfrom the highest places (WCC 185 and WC150) showed the highest similarity values.

In this study, Richness and Shannon indices were selected to estimate the fungal diversity. As shown in Table 2, there are significant differences (*p* < 0.05) among the samples collected from different sampling sites in the tomb. Among the 6 sampling sites, WC30 showed the highest richness and diversity, followed by WCC30 and WCC185, while WC150 was the lowest.

**Table 2:** Richness and Shannon indices estimates for fungal communities at different sampling sites from emperor Yang’s coffin chamber.

	Richness	Shannon
WCC30	14 ± 3a	2.34 ± 0.18b
WCC105	10 ± 1b	2.30 ± 0.06b
WCC185	13 ± 1a	2.42 ± 0.05b
WC30	17 ± 2a	2.64 ± 0.15a
WC80	10 ± 1b	2.06 ± 0.07c
WC150	9 ± 1b	1.89 ± 0.02c

The values are the means of the three replicates (mean ± SD). Values followed by different letters differ significantly (*p* < 0.05).

### Phylogenetic identification of fungal 18S rRNA genes

Phylogenetic analysis showed that 18 fungal genotypes obtained from the DGGE profiles (Table 3). The sequences showed highest 18S rRNA gene sequence similarity to the *Cordyceps*, *Fusarium*, *Harpochytrium*, *Emericellopsis*, *Volutella*, *Cladosporium*, *Stachybotrys*, *Trichoderma*, *Cochlonema* and two unknown fungal species (Table 3). Specifically, *Stachybotrys*, *Fusarium*, *Trichoderma* and *Cochlonema* were considered as the dominant fungal groups in Emperor Yang’s coffin chamber (based on the patterns and densities of DGGE bands: 12, 13, 14, 15, 16, 17 and 18).

**Table 3:** Phylogenetic identification of DGGE band recovered from the gel.

DGGE Band	Top BLAST search results (Similarity, %)	Query coverage (%)	NCBI gene accession no.
Band 1	*Cordyceps confragosa* (97.7%)	100%	MH521026
Band 2	*Fusarium* sp. F116 (100%)	100%	MN240475
Band 3	*Harpochytrium* sp. JEL290 (90.1%)	100%	KJ668051
Band 4	*Fusarium* sp. AHMF4 (100%)	100%	MN094111
Band 5	*Emericellopsis alkalina* (100%)	100%	NG062924
Band 6	*Volutella ciliata* (100%)	100%	AJ301966
Band 7	*Cladosporium bruhnei* (99.1%)	100%	MH879819
Band 8	*Fusarium* sp. F116 (99.7%)	100%	MN240475
Band 9	*Stachybotrys chartarum* (96.2%)	100%	KC787690
Band 10	Uncultured eukaryote (96.9%)	100%	LC150171
Band 11	*Fusarium* sp. F116 (99.7%)	100%	MN240475
Band 12	*Stachybotrys chartarum* (96.2%)	100%	KC787690
Band 13	*Fusarium equiseti* (99.7%)	100%	MF522214
Band 14	*Fusarium* sp. F116 (99.7%)	100%	MN240475
Band 15	*Trichoderma* sp. CAR_1 (97.4%)	100%	MH016738
Band 16	*Fusarium* sp. AHMF4 (99.4%)	100%	MN094111
Band 17	Uncultured fungal isolate (96.5%)	99%	KF741829
Band 18	*Cochlonema euryblastum* (97.7%)	100%	DQ520640

### Effect of the environmental factors on fungal community composition

**Figure 3:**
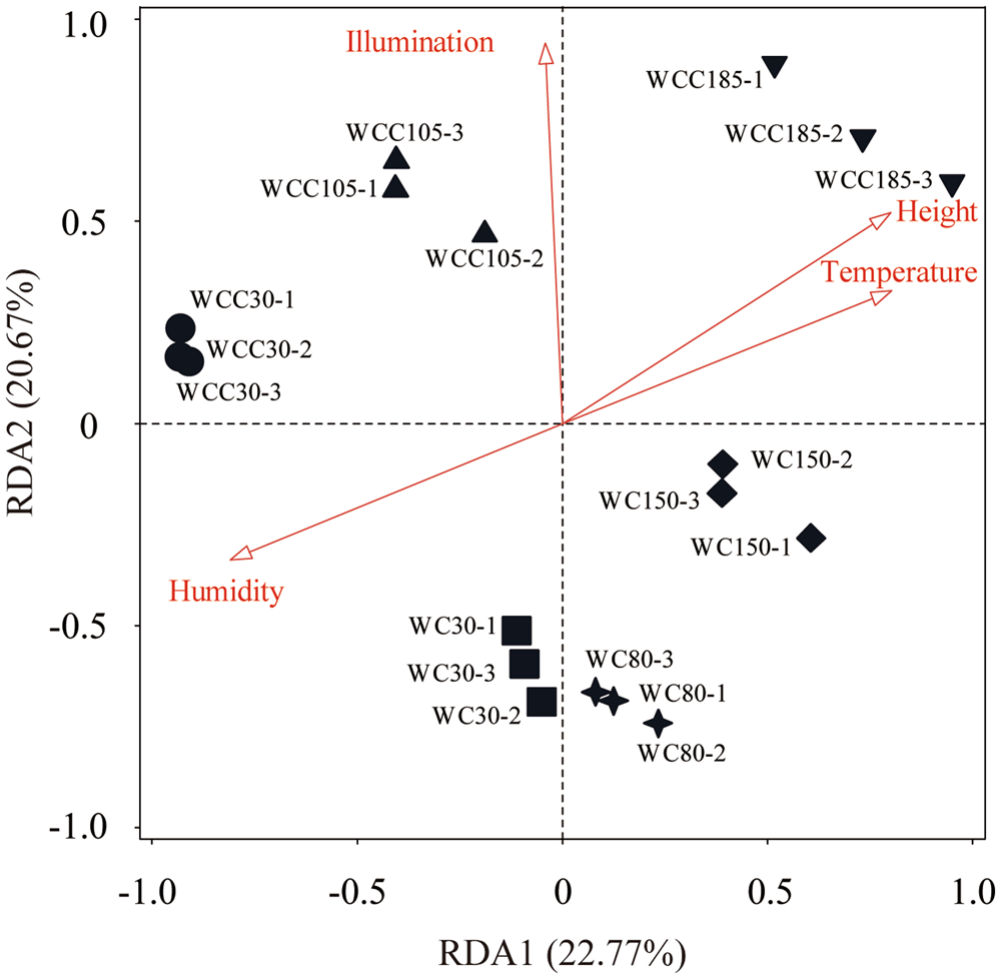
RDA ordination diagram of environmental factors in relation to samples. Environmental parameters are indicated by lines with arrows, and the samples are represented by different marks. WC means the points on the walls of passage in the coffin chamber, and WCC means on the walls of main room. The numbers refers to the height of the sampling points are 30, 80, 150, 105 and 185 centimeters above the floor of coffin chamber.

The result of DCA showed that the gradient length of the first axis was computed to be 2.90. According to this, RDA was used to analyze the correlation between fungal community composition and environmental factors. As shown in Fig. 3, the first two axes together described the 43.44% of the total variance of the community. Humidity had the greatest impact (Table S1), supporting 19.2% of the explained variance in the dataset, followed by illumination (18.3%), height (12.8%) and temperature (10.1%). This finding suggests that all the four environmental factors significantly affected fungal communities in emperor Yang’s coffin chamber. Furthermore, Spearman correlation analysis also revealed that environmental factors affected the fungal diversity and community composition (Table 4). Fungal richness and Shannon indices were positively related to humidity (*p* < 0.05).

**Table 4:** Statistical analysis of correlation coefficients between fungal diversity indices and environmental factors.

	Richness	Shannon
Height	−0.42	−0.35
Humidity	0.55*	0.64**
Temperature	−0.44	−0.38
Illumination	0.02	0.28

^*^p < 0.05; **p < 0.01, Black bold numbers indicate significant differences.

## Discussion

Important historical buildings with brick masonry or stone structure are often reported to be corroded. This predominantly occurs due to microbial-based activities, particularly in environments with higher moisture^18,19^. Emperor Yang’s coffin chamber is one of the most valuable buildings in China. Our previous study on this ancient remnant has revealed some bacterial-related groups that were associated with the tomb^20^, and managed to isolate a novel bacterial species, *Paenibacillus tumbae*^21^. In this study, we firstly investigated the fungal communities in the walls of Emperor Yang’s coffin chamber, and hypothesized that the existed diversity fungal species. The results, based on DGGE profile, showed that the dominant fungal genera were belonging to *Stachybotrys*, *Fusarium*, *Trichoderma* and *Cochlonema* (Fig. 2a and Table 3), findings in line with our hypothesis. This observation was consistent with what was previously reported with regards the biota thriving in historic and residential buildings. For example, in Northern Europe and North America only, it was estimated that between 20% and 40% of buildings were contaminated by indoor mold among which *Stachybotrys* sp., *Fusarium* sp., *Acremonium* sp. are the most predominant ones^22^.

*Stachybotrys chartarum* is known as “black mold” or “toxic black mold”, which is commonly found in soils and indoor buildings^23,24^, particularly in damp environments. In this study, DGGE band 12 showed the highest 18S rRNA sequence similarity with *S*. *chartarum*, demonstrating that this species indeed existed in the coffin chamber. Gutarowska *et al*.^25^ isolated *S. chartarum* from a wallpaper, and found that it had a great ability to produce organic acids that could significantly decrease pH. Although the direct effect of *S. chartarum* on biodeterioration of building material was not investigated, potential risks worth of attention due to the production of organic acids. In addition, previous studies also highlighted that this fungal species could synthesize mycotoxin such as trichothecenes^26^, and was associated with human health deterioration such as airway infections, bronchitis, asthma and extreme fatigue^23^.

*Fusarium* sp. was commonly recognized as a plant pathogenic fungi, and some species of this genus were often reported to induce plant diseases such as *F*. *wilt* and *F*. *oxysporum*^27,28^. These species are generally detected in tombs or buildings^29,30^. Our results showed that *Fusarium* spp. were also the dominant species in the coffin chamber, which were corresponding to DGGE band 2, 4, 6, 8, 11 and 14. *Fusarium* spp. demonstrated to have the ability to deteriorate stones and concrete, and even produce pigments^31,32^. Guglielminetti *et al*.^33^ found that *Fusarium* sp. is a key specie of the fungal community responsible for the deterioration of the indoor wall paintings (frescos) in the Monastery of St Damian in Assisi. Similar to *S. chartarum*, *Fusarium* spp. have recently emerged as a significant human pathogen^34^. So, the presence of *Fusarium* spp. in coffin chamber or buildings is becoming a potential threat for both humans and environment.

One more finding from this study is that *Trichoderma* (DGGE band 15) was the dominant fungal species on the walls of Emperor Yang’s coffin chamber, observation very well aligned with the findings of Rao *et al*.^35^. Species of *Trichoderma* genus are versatile in nature with varying activities, for example, they are often related to suppress soil-borme diseases and/or produce celluases and xylanases^36,37^. They are also commonly found in indoor environments^38,39^, and considered as one of the indicators of moisture-damaged building materials^40^. *Trichoderma* and *Fusarium* have been identified in Godoy’s House, a historical building^41^. Samson *et al*.^42^ highlighted that *Stachybotrys* spp. and *Trichoderma* are likely to arise when the water activity (a_w_) is greater that 0.85. In this study, DGGE band 15 showed the highest densities in samples WCC105 and WC30, respectively, where the air humidity was 88%, observation in line with previous studies^42^. In addition, *Trichoderma* species are not known to be harmful to humans, but there is accumulating evidence that a few species may be infectious for humans^43,44^. Particularly, we also found the dominance of *Cochlonema*, corresponding to DGGE band 18, in emperor Yang’s coffin chamber; however, to our knowledge, there is no available report that associates this specie with indoor environment, building or human disease. In this study, we also identified the presence of *Cordyceps*, *Harpochytrium*, *Emericellopsis*, *Volutella*, *Cladosporium* and two unknown fungal species, most of which were previously found in buildings. In this study, we could not discuss them in detail due to their low relative abundances.

Previous studies have shown that fungal community composition inside the buildings is driven by a wide range of environmental factors, such as humidity, water content or moisture, sampling locality and building function^45,46^. In this survey, we highlighted that fungal communities in the walls of Emperor Yang’s tomb were predominantly affected by humidity, illumination, air temperature and height of sampling sites (Fig. 3), findings generally in accordance with previous studies. One of the most mentioned factors that affects indoor fungal communities is humidity or moisture. For example, Doll and Burge^47^ reported that fungal species and their growth is shown to increase with moisture. In our investigation, the results showed a higher correlation between humidity and the value of fungal richness and diversity indices (Table 4), which was partly in accordance with previous studies. Furthermore, Hegarty *et al*.^48^ determined the gene expression of dust fungal communities at water activity (a_w_) levels of 0.5, 0.85 and 1.0, the results showed that the metabolic processes of fungal communities at a_w_ level of 0.5 only referred to cell maintenance, but displayed more diverse secondary metabolic processes at 1.0. These results totally highlighted that humidity or moisture have a pronounced impact on controlling fungal growth. Actually, moisture is considered as the only limiting factor for fungal growth in buildings^45^. As we know, fungi need adequate temperature, nutrients and water to grow. Usually, building materials, even fiberglass fabric coverings, could provide with nutrients such as cellulose and starch for fungal growth^49,50^, and some phototrophic microorganism such as cyanobacteria and lichens could also supply with organic nutrients for bacteria and fungi^51,52^. As for temperature, in this study, this factor ranged between 24.07–25.47 °C, suitable for fungal growth^53,54^. Although the result of RDA revealed the significant influences of temperature, height of sampling site and illumination on fungal communities, here, we did not discuss their effects because they were directly or indirectly related to humidity (Table S2).

Particularly, RDA also highlighted that illumination shaped the fungal community composition (Fig. 3). However, it is widely accepted that the illumination or light in general is not the necessary for the growth of fungal species. Lichen model can be used to reasonably explain this phenomenon^51,55^. Small amounts of nutrients in rocks or bricks were not enough to support vigorous growth of fungal communities, but the colonization of photosynthetic microorganisms, such as algae and cyanobacteria, obviously improve the nutrient conditions due to their abilities to both photosynthesize and fix atmospheric nitrogen. The consequence of this was that more and more fungal communities could colonize on the surface of rock or brick. So, in the future work, we should also take into account the communities of algae and cyanobacteria, which will provide more information for us to understand the microbes on rocks. On the other hand, illumination has an impact to temperature and humidity, which will further affect fungal communities.

## Conclusion

The fungal community structure developed at different points in emperor Yang’s coffin chamber was investigated to understand the plausible links between fungal community individuals and biodeterioration, and their potential responses to various environmental factors. 18 dominant 18S rRNA gene sequences were recovered from DGGE gel, the majority of which belonged to *Stachybotrys*, *Fusarium*, *Trichoderma* and *Cochlonema*. Environmental factors such as humidity, temperature, sampling height and illumination significantly influenced the fungal community composition in emperor Yang’s coffin chamber. Humidity was the factor that was most significantly (positively) related to fungal richness and Shannon indices. This provides with useful information to further understand the fungal species emerge in a tombs of such kind, and reveals the key environmental factors that shape fungal diversity and community composition.

## Materials and methods

### Sampling and physical determination

The bottom of Emperor Yang’s coffin chamber is 2.95 m below the ground surface, which means the chamber is embedded in the soil (Fig. 1). Because of the temporary protection shed, the upper part of the wall in the main room of coffin chamber is illuminated but the lower part of it lacks light. According to the different degree of brightness, six measuring points were selected, i) on the walls from the passage, and ii) from the walls the main room of the coffin chamber. The three points on the wall of passage were sampled at the height of 30 cm (WC 30), 80 cm (WC 80), 150 cm (WC 150). Another three points on the wall of main room were sampled at the height of 30 cm (WCC 30), 105 cm (WCC 105), 185 cm (WCC 185), as shown in Fig. 1. The environmental characteristics at these six sampling points was measured by automatic temperature and humidity recorder (T&D Corporation, Japan) with precision of ±0.1 °C or ±0.1%, respectively. The levels of illumination were assessed using three degrees that was high, low and very low. The dust on wall surface was also sampled at these six points, each point was tested in triplicates. Dust samples were scratched and collected aseptically, and then were kept at −20 °C for molecular analysis.

### DNA extraction

Microbial DNA genomes of 0.5 g dust sample from various positions was extracted using a FastDNA® SPIN Kit for soil (MP Biomedicals, USA) according to the manufacturer’s instructions. The extracted DNA was dissolved in 50 μl TE (Tris-EDTA) buffer, quantified by spectrophotometer and stored at −20 °C until further use. Extraction was carried out within 24 hours after sampling.

### Denaturant gradient gel electrophoresis (DGGE) analysis

The primer set used was FF390 (5′-CGA TAA CGA ACG AGA CCT-3′) and FR1 (5′-AIC CAT TCA ATC GGT AIT-3′) with a GC-clamp (5′-CCC CCG CCG CGC GCG GCG GGC GGG GCG GGG GCA CGG GCC G-3′) at 5′ end^56^ was used to amplify ~390 bp fungal 18 S rRNA gene fragments of different samples in the coffin chamber. A DCode Universal Mutation Detection System (Bio-Rad, USA) was used for DGGE analysis. Approximate 150–250 ng PCR amplicons from each sample were electrophoresed on a 8% acrylamide- bisacrylamide gel, with 45% to 75% denaturant at 100 V for 10 h in 1×TAE running buffer at 60 °C. The gels were stained for 20 min with SYBR Green I nucleic acid gel stain (1:10000 dilution) (Invitrogen, USA). The gels were visualized and digitalized by using a Gel DocTM EQ imager (Bio-Rad, USA) combined with Quantity one 4.62 (Bio-Rad, USA). The representative bands were excised, left overnight in 25 μl Milli-Q water, reamplified and run again on the DGGE system to ensure purity and correct mobility of the excised DGGE bands. Correct PCR products were purified using the QIAquick PCR Purification kit (QIAGEN) before cloning.

### Cloning, sequencing and phylogenetic analysis

The purified PCR amplicons of the excised DGGE bands were cloned into a pMD18-T vector (TaKaRa, Japan) and transformed into *Escherichia coli* DH5*α* competent cell. Six random clones containing correct gene size for each DGGE band were sequenced by Invitrogen Sequencing Department in Shanghai. DNASTAR software package was used to manually check and compare the clone sequences. One representative clone sequence with high quality after sequence comparison from each band was used for phylogenetic analysis.

Together with the top three BLAST hits of homologous gene sequences, the gene sequences from cultured and well characterized species in Genbank, the DGGE band sequences were used to build a basic phylogenetic tree by the neighbor-joining method using the software package of MEGA 7.0 version (Molecular Evolutionary Genetics Analysis)^57^. The tree topology was further evaluated by different algorithms including Minimum Evolution and Maximum Parsimony. The phylogenetic relationships of fungal 18S rRNA gene sequences to the closest homolog in the GenBank were then inferred.

### Statistical analysis

Statistical analysis was carried out with the software package SPSS 13.0 for Windows. Data were expressed as means with standard deviation (SD). Mean separation was conducted based on Tukey’s multiple range test. Statistically significant differences were considered at *p* < 0.05. DNA fingerprints obtained from the fungal 18S rRNA gene banding pattern on the DGGE gels were photographed and digitized using Bio-Rad’s Quantity One software (Version 4.62). Shannon index (*H*) is calculated based on the mathematical formula: *H* = −∑(p_i_)(lnp_i_), which pi stands for the proportion of species i relative to the total number of species. Richness is calculated according to band number of each sample. Spearman correlation was used to determine the relationships between diversity indices and environmental factors. A detrended correspondence analysis (DCA) was performed for the response variable data to estimate the heterogeneity through the length of the community composition gradients. After confirming the gradient length of axis 1, redundancy analysis (RDA) was performed to arrange fungal communities based on physical properties using Canoco for windows (version 5.0)^58^.

## Supplementary information

Supplementary information

## Data Availability

The authors agree to make data in this manuscript available to readers.
