# Supplementary material for: Humidity governs the wall-inhabiting fungal community composition in a 1600-year tomb of Emperor Yang
Source: Sci Rep. 2020 May 21;10:8421. doi: 10.1038/s41598-020-65478-z (PMC7242468; doi:10.1038/s41598-020-65478-z)
Supplement: Supplementary file 1 — Supplementary information [file 41598_2020_65478_MOESM1_ESM.pdf]

Humidity governs the wall-inhabiting fungal community composition in a 1600-year tomb of Emperor Yang

Yonghui Li[a,b1], Zhi Huang[c], Evangelos Petropoulos[d], Yan Ma[a], Yang Shen[a,b]

a. School of Architecture, Southeast University, 210096 Nanjing, PR China

b. Key Laboratory of Urban and Architectural Heritage Conservation (Southeast University), Ministry of Education, 210096 Nanjing, PR China

c. Key Laboratory of Agricultural Environmental Microbiology, Ministry of Agriculture, College of Life Sciences, Nanjing Agricultural University, 210095 Nanjing, PR China

d. School of Engineering, Newcastle University, Newcastle upon Tyne, NE1 7RU, UK

1 Corresponding authors: Yonghui Li, E-mail: liyonghui@seu.edu.cn

Supplementary information

Table S1 The explains and contribution of each environmental factor to fungal community composition

	Explains %	Contribution %	pseudo-F	*p*
Humidity	19.2	31.7	3.8	0.002
Illumination	18.3	30.3	4.4	0.002
Height	12.8	21.2	3.6	0.006
Temp	10.1	16.8	3.3	0.008

Table S2 Pearson correlations between diversity indices and environmental factors

	Height	Humidity	Temperature	Illumination
Height	-			
Humidity	-0.88**	-		
Temperature	0.94**	-0.85**	-	
Illumination	0.41	-0.08	0.22	-

* p<0.05; ** p<0.01

Table S3 Comparison of RDA and CCA in explaining fungal community composition

	Explained variation of the first two axes (%)	Explains of each environmental variable (%)			
		Humidity	Illumination	Height	Temperature
RDA	44.44	19.2	18.3	12.8	10.1
CCA	44.77	21.4	19.0	11.0	12.8

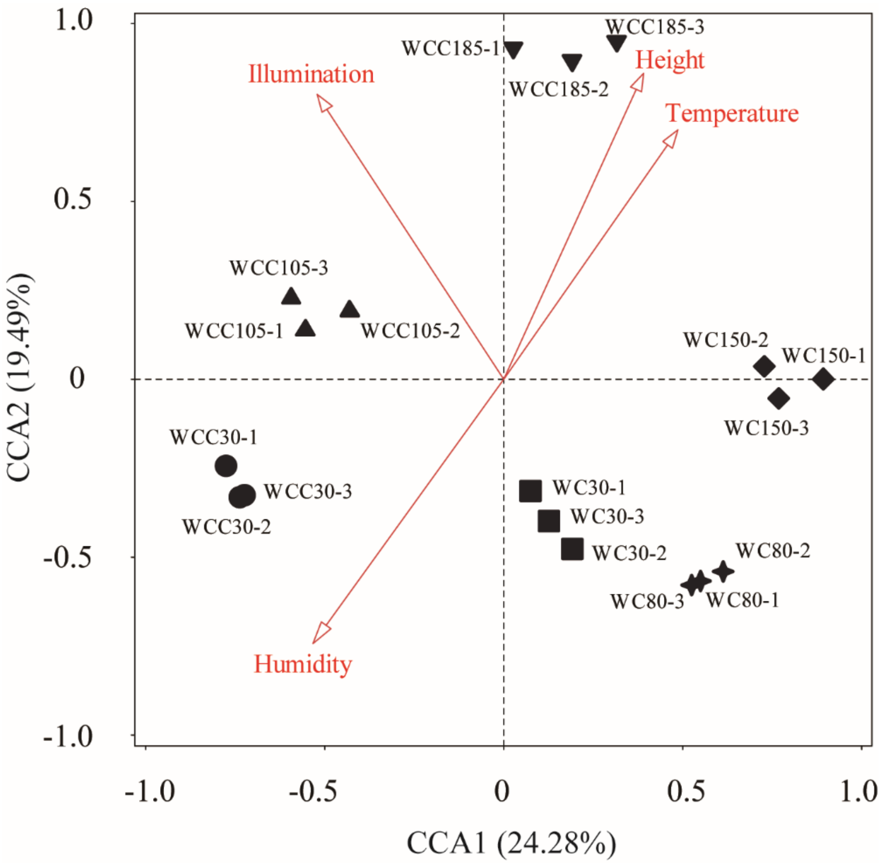

Fig. S1 Canonical correspondence analysis (CCA) ordination diagram

Environmental parameters are indicated by lines with arrows, and the samples are represented by different marks. WC means the points on the walls of passage in the coffin chamber, and WCC means on the walls of main room. The numbers refers to the height of the sampling points are 30, 80, 150, 105 and 185 centimeters above the floor of coffin chamber.
